# Hanhart syndrome: hypoglossia-hypodactylia syndrome

**DOI:** 10.11604/pamj.2019.32.213.17493

**Published:** 2019-04-29

**Authors:** Ipek Guney Varal, Pelin Dogan

**Affiliations:** 1 Department of Pediatrics, Division of Neonatology, University of Health Sciences, Bursa Yüksek Ihtisas Teaching Hospital, Bursa, Turkey

**Keywords:** Hanhart syndrome, hypoglossia-hypodactylia, Turkey

## Image in medicine

Hanhart syndrome is a congenital disorder that causes an undeveloped tongue and malformed extremities and fingers. Small mouth, short or incompletely developed tongue (hypoglossia), absent or shortened fingers and/or toes, jaw abnormalities such as micrognathia, retrognathia or partially missing mandible (lower jaw), high-arched, narrow, palate, absent or unusually formed arms and/or legs. If the tongue and/or mouth are affected, this can worsen feeding difficulties that are already present due to the craniofacial abnormalities. A diagnosis of Hanhart syndrome is typically made based on the presence of characteristic signs and symptoms. To date, no specific disease-causing genes have been identified. We present the case of a 3000 g male infant who was born at 39 weeks' gestation to a 42-year-old gravida 2 para 2 mother via cesarean section. At the physical examination he was noted to have adactyly at two hands, micrognathia, incompletely developed tongue and high-arched palate. Tongue movements were inadequate because of the small size. When we looked at the pregnancy history the mother took thyroid drugs cause of hypothyroidism after thyroid surgery and there was no family history of congenital anomalies or consanguinity. Due to feeding difficulties he stayed at neonatal intensive care unit. Cranial and abdominal ultrasonographyic examination of the infant was otherwise normal. The infant was discharged home on full oral feedings on day 6.

**Figure 1:**
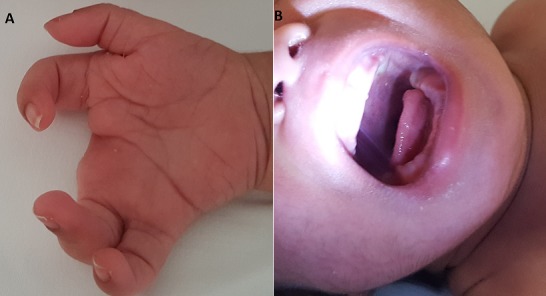
A) adactyly at two hands; B) micrognathia, incompletely developed tongue and high-arched palate

